# The effects of a controlled worksite environmental intervention on determinants of dietary behavior and self-reported fruit, vegetable and fat intake

**DOI:** 10.1186/1471-2458-6-253

**Published:** 2006-10-17

**Authors:** Luuk H Engbers, Mireille NM van Poppel, Marijke Chin A Paw, Willem van Mechelen

**Affiliations:** 1 Department of Public and Occupational Health and EMGO-institute, VU University Medical Center, Van der Boechorststraat 7, 1081 BT, Amsterdam, The Netherlands; 2 Body@Work, Research Center on Physical Activity, Work and Health. Institute for Research in Extramural Medicine of the VU University Medical Center (EMGO-institute) and TNO Quality of Life, The Netherlands

## Abstract

**Background:**

Eating patterns in Western industrialized countries are characterized by a high energy intake and an overconsumption of (saturated) fat, cholesterol, sugar and salt. Many chronic diseases are associated with unhealthy eating patterns. On the other hand, a healthy diet (low saturated fat intake and high fruit and vegetable intake) has been found important in the prevention of health problems, such as cancer and cardio-vascular disease (CVD). The worksite seems an ideal intervention setting to influence dietary behavior. The purpose of this study is to present the effects of a worksite environmental intervention on fruit, vegetable and fat intake and determinants of behavior.

**Methods:**

A controlled trial that included two different governmental companies (n = 515): one intervention and one control company. Outcome measurements (short-fat list and fruit and vegetable questionnaire) took place at baseline and 3 and 12 months after baseline. The relatively modest environmental intervention consisted of product information to facilitate healthier food choices (i.e., the caloric (kcal) value of foods in groups of products was translated into the number of minutes to perform a certain (occupational) activity to burn these calories).

**Results:**

Significant changes in psychosocial determinants of dietary behavior were found; subjects at the intervention worksite perceived more social support from their colleagues in eating less fat. But also counter intuitive effects were found: at 12 months the attitude and self-efficacy towards eating less fat became less positive in the intervention group. No effects were found on self-reported fat, fruit and vegetable intake.

**Conclusion:**

This environmental intervention was modestly effective in changing behavioral determinant towards eating less fat (social support, self-efficacy and attitude), but ineffective in positively changing actual fat, fruit and vegetable intake of office workers.

## Background

Lifestyles in Western industrialized countries are characterized by a decreasing level of physical activity [1-3], a high energy intake and an overconsumption of (saturated) fat, cholesterol, sugar and salt [4]. According to the Food consumption survey of 2003 (5), among young adults (age 19–30) in the Netherlands, only 2% meet the recommendation for fruit intake (i.e. 150 gram per day) and 0% meets the recommendation for vegetable intake (i.e. 134 gram per day). Regarding saturated fat intake only 8% of the young adults meets the recommendation for saturated fat intake (i.e. 10 energy% saturated fat of total energy intake) [5].

A healthy diet (low saturated fat intake and, high fruit and vegetable intake) has also been found important in the prevention of health problems, such as some types of cancer and cardiovascular disease (CVD) [6-8]. Moreover, in a review to evaluate the evidence regarding diet and CVD prevention, substantial evidence was found that diets, containing unsaturated fat and an abundance of fruits and vegetables, offer protection for CVD. However, the authors mentioned that such diets have to coincide with regular physical activity, not smoking and maintaining a healthy body weight [9]. Nevertheless, stimulating healthy food habits seem to be important.

Worksites are an effective channel to promote healthy food habits among employees by means of comprehensive worksite health promotion programs (WHPP's), because they provide access to a large proportion of the adult population and people spend a great deal of their time at the worksite. In many WHPP's, traditional methods (i.e. individual counseling, education, group sessions) to increase knowledge and skills are used to stimulate healthy behavior [10-13]. However, currently more and more attention is drawn to changing the physical (worksite) environment [14-17] by creating opportunities and by removing barriers to facilitate healthy behavior. It is now assumed that environmental strategies should at the least be incorporated in traditional WHPP's to achieve greater behavioral changes and to reach a wider audience. In a literature review, specifically [18] focusing on the effectiveness of WHPP's with environmental components only a few of such programs was found. Nevertheless, it was concluded that there was relatively strong evidence for the effectiveness of these WHPP's on fat, fruit and vegetable intake. However, all studies reviewed were multi-component studies. So it was impossible to draw solid conclusions about the contribution of the environmental components to the effects of these interventions.

Therefore, a worksite intervention (i.e. FoodSteps) solely consisting of relatively modest environmental changes was developed to stimulate physical activity, but also healthy food habits of office-workers. The purpose of this study is to present the effects of this intervention on determinants of dietary behavior and on self-reported fat, fruit and vegetable intake.

## Methods

### Study design and population

In this controlled longitudinal trial, two different government companies in The Hague (the Netherlands) were used: one intervention and one control company. These worksites were chosen because of the similar job-descriptions of the employees. The inclusion criteria for participating in the study were; (1) office worker, (2) the ability to climb the stairs, (3) a body mass index (BMI) ≤ 23 and (4) a contract for at least the duration of the intervention. In a review on the public health burden of obesity of Visscher et al [7], a number of studies was included that described an increased risk for CVD, all cause mortality, type 2 diabetes mellitus and stroke with a BMI ≤ 22.5 (kg/m^2^) in women and a BMI ≤ 23 (kg/m^2^) in men. In order to select a population at higher risk for disease associated with overweight, the inclusion criterion of a BMI ≤ 23 was applied in our study. Subjects who were pregnant or became pregnant during intervention year, or had severe cardiovascular/musculoskeletal disorders were excluded. Employees received a leaflet by company internal mail system in which they were asked to participate in the study and they had to return a written reply form to be included in the study. On the reply form a number of screening questions (including self-reported body weight and body height) had to be filled out. A written informed consent was obtained from the subjects and this study had the approval from the medical ethics committee of the VU University Medical Center.

The questionnaires were distributed among subjects at both worksites at baseline (October 2003), at three months (April 2004) and 12 months (November 2004).

### Intervention

The FoodSteps intervention consisted of two parts, one part focusing on food (i.e. stimulating healthy food choices) and one on physical activity (i.e., stimulating stair-use). The food-intervention took place over 12 months (January 2004–December 2004) in the company canteen of the intervention company and mostly consisted of placing informational sheets near food products, to stimulate healthier food choices. Every four weeks one group out of six groups of products was chosen to be highlighted. Each group was repeated once during the year. On the informational sheets the caloric (kcal) value of a product was translated into the number of minutes to perform a certain (occupational) activity (e.g. climbing stairs, having a meeting or doing a lunch-walk). The product-groups were: (1) dairy products (i.e. milk, yogurt and other deserts) (2) warm snacks, (3) fruit-vegetables-salads, (4) cold ready-to-eat sandwiches (including fillings) (5) sandwich fillings (i.e., high and low fat cold meats and cheeses and several sweets) and (6) pastry. On three vending machines similar information sheets were placed, on which the snacks (candy bars, crisps, [diet] soda's) offered in the machines were highlighted. The sheets on the vending machines were not changed during the intervention year. Additionally, an information stand was placed in the canteen with brochures and leaflets on healthy food, blood pressure and cholesterol. Finally, every two months during one day a week a buffet with healthy products was offered to the customers of the company canteen.

### Outcome measures

#### Psychosocial determinants of behavior

Psychosocial determinants of eating more fruit, vegetables and less fat were measured applying the 'attitude-social influence- (self-)efficacy model' (ASE model) [21,22]. All items were measured using a 7-point Likert-scale. Each subject had to fill out to what degree he/she agreed with a number of statements regarding eating less fat or more fruit and vegetables. Attitude was measured with one item 'Do you think that eating less fat takes a lot of effort, or not? (-3 = a lot of work; +3 = no work at all).' Social influence was measured by the perceived support from colleagues 'Do your colleagues in general stimulate you to eat less fat?' (-3 = absolutely not; +3 = yes, absolutely). Self-efficacy was measured by one item 'Do think it would be easy to eat less fat (or more fruit, vegetables) at work, if you really wanted to?' (-3 = very difficult; +3 = very easy). Finally, intention was measured with one item 'Do you intend to eat less fat within the coming month?' (-3 = absolutely not; +3 = yes, absolutely). Determinants regarding fruit and vegetable consumption were measured in a similar manner.

#### Fruit and vegetable consumption

The validated Short Fruit and Vegetable questionnaire was used to measure fruit and vegetable consumption. This questionnaire consists of 10 questions: 6 about fruit consumption and 4 about vegetable consumption [19]. Subjects were asked to mark on how many days in a normal week (over the last month) they had consumed citrus fruit, other fruit, unsweetened fruit juice, heated vegetables and raw vegetables. They were also asked to mark the number of serving spoons (vegetables), pieces (fruit) and glasses (juice) they had consumed on a day that fruit or vegetables were consumed. In calculating the mean daily vegetable consumption in grams, a serving spoon was standardized as 50 grams.

#### Fat consumption

In this study the validated Fat list [20] was used to measure fat intake. This list consists of 35 questions covering 19 (categories of) food items. Subjects were asked about the frequency of consuming certain food items during the last month and (if applicable) additional questions on quantity or kind of product were asked. For each of the 19 categories of food items a fat score, ranging from zero points (lowest fat intake) to a maximum of five points (highest fat intake), was determined. This fat score equals a certain amount of daily fat intake, for instance: a fat score of 4 points for milk equals an intake of 13–16 grams of fat per day and a fat score of 1 point equals 1–4 grams per day. A total fat score (range 0 – 60) could be calculated by adding up the 12 fat scores. Fat scores obtained from products in hot meals were excluded (7 items), in an attempt to limit the contribution of fat from food items consumed outside the worksite (e.g., at home).

### Covariates

The following data were collected by questionnaire: the highest achieved level of education, age, smoking (yes/no), number of alcoholic units per week, hours per week at the office, whether or not following a diet, whether or not being a regular visitor of the company canteen (at least once week purchasing food in the canteen) and whether or not taking lunch to work every day of the week. Additionally, as a part of the study, subjects were invited to attend a physical examination at all follow-ups where among other variables, body height (cm) and body weight (kg) were measured with subjects in underwear. The Body Mass Index (BMI) as measured at baseline was also used as a covariate in this study. BMI was calculated by dividing body weight (kg) by body height (m) squared (= kg/m^2^).

### Statistical analysis

Both the short-term (3 months) and the long-term (12 months) effect of the intervention were analyzed by multivariate linear regression analysis. In this analysis the outcome at respectively 3 and 12 months was corrected for the baseline value. The regression-coefficient of the group allocation (0 = control worksite, 1 = intervention worksite) variable reflects, the difference in change over time between worksites in the outcome variable. Linear regression analysis excludes subjects with missing data. Only subjects with baseline data and data on at least one follow-up were included in the analysis. Baseline values that differed (according to independent t-test) between intervention and control subjects at baseline, as well as a set of predefined variables (i.e. gender, age, BMI, alcohol consumption and smoking) were checked as possible confounders. As possible effect modifiers were considered baseline data on: gender, BMI, whether or not taking lunch to work, being a regular visitor of the company canteen, smoking and alcoholic intake. Effect modification was defined as a significant (p < 0.10) interaction term between the group allocation variable and the variable of interest.

## Results

### Subjects

**Figure 1:**
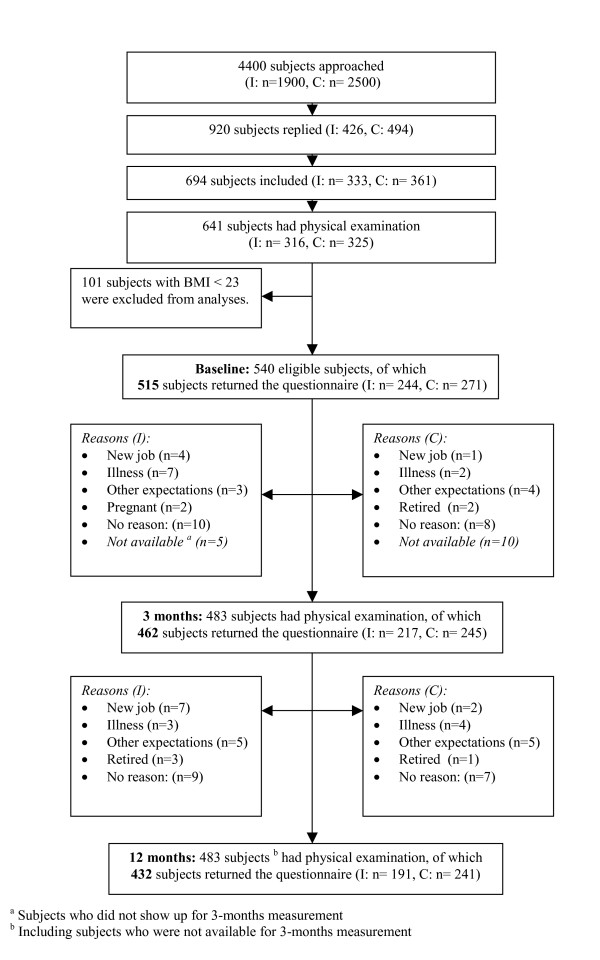
Flow-chart of the intervention (I) and control (C) subjects in the trial.

In figure 1, the number of subjects in the trial is shown. At both the control and intervention worksite a combined total of 4400 employees were approached and 20.9% (n = 920) expressed their interest to participate in the study. Based on the information on the reply-forms 694 subjects were included, of who 641 showed up for the physical examination at baseline. After analyzing the data of the physical examination at baseline, the results showed that a number of 101 subjects had a BMI < 23. These subjects were excluded from analysis. Of the remaining subjects, eventually, baseline questionnaire data was obtained from 515 subjects.

Although, a higher number of included subjects (about 900) was intended, with a power of 0,8 en alpha of 0,05, a difference of about a half (0.42) piece of fruit and 20.7 grams of vegetables can still be demonstrated with a total number of 515 participants.

Questionnaire return-rates in the intervention site were 88.9% and 78.3% and in the control site 90.4% and 88.9%, at 3 and 12 months respectively. The baseline demographics of the total population are described in Table 1. In both worksites more men than women were included in the study. This was in accordance with the general gender distribution in both worksites (approximately 35.0% female). The subjects in the control worksite were significantly (p < .01) more hours per week at the office than those at the intervention worksite, and the intervention worksite had significantly more regular visitors to the company canteen (p < .01).

### Psychosocial determinants of dietary behavior

Table 1 shows the baseline mean scores on the behavioral determinants regarding eating less fat, and more fruit and vegetables. At three months, social support towards eating less fat showed a significant difference in change (diff. = 0.34, 95%CI: 0.08; 0.60) in favor of the intervention group (Table 2). This effect was due to an increase in this group, compared to no change in the control group. At 3 months, self-efficacy towards eating less fat showed a significant difference in change (diff.: -0.35, 95%CI: -0.60; -0.09) in favor of the control group, due to a decrease in the intervention group. This effect was also found at 12 months (diff.: -0.44 95%CI: -0.70; -0.18). Finally, at 12 months, the attitude towards eating less fat showed a significant difference in change (diff. = -0.31, 95%CI: -0.05; -0.58), in favor of the control group, this could again be attributed to a small decrease in the intervention group.

In addition, a significant negative interaction was found with BMI at baseline. This can be interpreted as an increasing intervention effect regarding the attitude to eat less fat at work for subjects with a higher BMI at baseline. No significant effects on any of the other psychosocial determinants were found.

### Fruit and vegetable intake

Table 1 shows the median fruit intake and mean vegetable intake at baseline for the intervention and control group. Regression analysis showed no significant difference in change between the intervention and the control group in fruit and vegetable intake (Table 3) at 3 and 12 months. Adjusting for the predetermined confounders did not change the results.

### Fat intake

In Table 1 the mean baseline fat intake by gender for the intervention and control group are shown. Regression analysis showed no significant differences (Table 3) in change between the intervention and the control group in fat intake at 3 or 12 months. Adjusting for pre-determined confounders did not change the results. At 3 months, an interaction was found with whether or not a subject took lunch to work. In the intervention group, the subgroup of subjects who did not take their lunch to work every day of the week at baseline had a significantly higher fat intake (diff: 0.77 fat-points, 95%CI: 0.09; 1.45), compared to those in the control group. Although not significantly, fat intake decreased (diff: -0.25 fat-points; -1.02; 0.52) for subjects in the intervention group who brought their lunch to work every day of the week, compared to those in the control group. No significant interactions were found at 12 months.

## Discussion

The purpose of this study was to analyze the effects of a worksite environmental intervention on determinants of dietary behavior regarding eating more fruit and vegetables and eating less fat and on actual (self-reported) fat, fruit and vegetable intake.

The results of this controlled trial showed that this environmental intervention only had a modest effect on determinants of dietary behavior. A significant effect was found on the perceived social support from colleagues regarding eating less fat. This determinant significantly increased at the short-term and borderline significant at long-term. However, also counterintuitive effects were found. First, at 12 months the attitude toward eating less fat decreased in the intervention group and decreased even more for subjects with a higher BMI at baseline. Second, self-efficacy towards eating less fat at work decreased significantly in the intervention group. The intervention was ineffective in significantly increasing fruit, vegetable intake and decreasing fat intake of the intervention group. An interesting finding was, however, that in the intervention group at short term the subgroup of workers who did not take their lunch to work every day significantly increased their fat intake compared to those in the control group.

Just as in our study, in a controlled trial of Steenhuis et al [23] a similar lack of results on self-reported fat, fruit and vegetable intake was found. In that trial, the effectiveness of two environmental programs in worksite cafeterias of seventeen worksites was evaluated. In the first environmental program a larger variety of low fat products, and fruit and vegetable were offered in the canteen. In the second program low fat products were labeled. In contrast to our environmental intervention, both programs were combined with an educational program and were compared with just an educational program alone and a control condition. No intervention effects of the combined intervention programs were found on self-reported fruit, fat and vegetable intake. In addition, in the Steenhuis study, no effects were found also on determinants of behavior regarding eating less fat, and more fruit and vegetables. In contrast, our intervention was effective in significantly increasing social support regarding eating less fat. However, in our study as a result of the intervention the attitude and self-efficacy scores became more negative. This can be interpreted as a re-evaluation of their food habits by the subjects in the intervention group as a result of the food information provided in the company canteen. Because of this intervention the subjects might have perceived it as more difficult to eat less fat (at work), in contrast to previous beliefs.

Other worksite health promotion programs (WHPP's) did show positive results on self-reported fruit-vegetable and fat intake. These trials [24-29] were included in our review on the effectiveness of WHPP's with environmental components [18]. It concerned trials that combined education, counseling or other individual strategies, with environmental changes. These environmental changes mostly consisted of extending the availability of healthy products and food labeling. Besides the fact that these trials applied combined interventions, another major difference with our study was that in these studies a more heterogeneous (blue and white collar) population was approached.

This difference in study population is an important point that might explain our poor results. In our study a primarily white-collar and highly educated population participated. White-collar populations are known to have in general more favorable food patterns (i.e. they eat more fruit-vegetables and less fat) [30] Therefore, a possible ceiling effect might have prevented the fruit and vegetable intake to increase, which might explain the slight decrease in mean vegetable intake observed at both worksites. When comparing vegetable intake in our population at baseline (i.e. 150 to 165 grams per day) to the general Dutch vegetable consumption recommendation (i.e. at least 150–200 grams of vegetables per day), it can be concluded that the baseline values were already relatively adequate, leaving little room for improvement. This seems a valid argument, when comparing these baseline values to the mean vegetable intake in the Dutch population, which was 134 grams per day in 1997 [5]. Baseline median fruit intake values in our study were also relatively high, with 1.8 to 2 pieces of fruit per day for the men and women, respectively. These figures correspond with the Dutch fruit intake recommendation (i.e. two pieces of fruit per day)[5]. Another contributor to possible ceiling effects in our study was the fact that a year before the intervention began; the canteen management had already changed their policy towards a healthier diet in the company canteen. For example, some 'bad' snacks were sold on only one day of the week and all 'bad' snacks were made more expensive. In contrast, fruit and vegetables were subsidized. This policy change at the intervention company should be regarded as a 'natural' environmental co-intervention.

Another explanation for the lack of positive results could be that in our study the same questionnaires as in the study of Steenhuis et al [23] were used. However, these questionnaires were not specifically developed to measure fruit-vegetable and fat intake in worksite canteens. By excluding the fat items regarding hot meals that are generally consumed at home, an attempt was made to limit the contribution of products consumed at home to the total fat score. In addition, our intervention focused also on vending machine products, but the questionnaire did not include questions on this issue. Nevertheless, these questionnaires were used to measure fruit-vegetable and fat intake, because of a lack of a validated short food frequency questionnaire, which are applicable to measure Dutch worksite food patterns.

A weak point in this study was that a relatively large proportion of the study population was not a regular visitor to the company canteen (about 40%). Because of this, the food intervention did not have the full impact it could have had. However, at follow-up no interaction was found between whether or not being a regular visitor to the canteen, and fruit-vegetable and fat intake. Also, the food intervention might have been too modest to sort any effect. As mentioned in the method section, only one product group at the time was highlighted by means of larger information sheets near the products included in the selected group. No information was put directly on the products and no clear-cut distinction between healthy or unhealthy products was made (for instance labeling products with either red or green colors), like in a study of Larsson et al [31]. Larsson et al used a food-marking symbol (the 'Green Keyhole') to make it easier for consumers to select low-fat and high fiber alternatives. This symbol was used on products that were an alternative to high-fat or low-fiber products. Perhaps in our study a comparable and a more obvious distinction between products should have been made. Instead of focusing on all products within pre-selected product groups (and one contrasting unhealthy alternative), the focus of the intervention should have been more on giving information solely on more healthy products. In our study it was hypothesized that, when giving information about the caloric value of a healthier product and the unhealthier alternative (e.g., high and low fat cheese), the subjects would choose a healthier alternative more often.

A limitation of this study might be the fact that no randomization was performed. Bias introduced by possible differences between worksites, might have been prevented if a randomization at the level of the individual could have been performed. However, due to the nature of the intervention this kind of randomization was not possible. Moreover, the main reason for not performing randomization at the level of the worksite was that, at the moment that the FoodSteps research proposal was approved, one worksite had already agreed to participate. In order to speed up the preparations of the intervention, this worksite was chosen as the intervention worksite. During this preparation period the control worksite still had to be found.

## Conclusion

In conclusion, this relatively modest environmental intervention was effective in significantly changing behavioral determinants towards eating less fat (social support, self-efficacy and attitude), but ineffective in significantly changing actual fat, fruit and vegetable intake of office workers. Negative changes in attitude and self-efficacy towards eating less fat at work were found. In future research it needs to be investigated if the food habits of employees can be changed by a more intensive environmental intervention.

## Competing interests

The author(s) declare that they have no competing interests.

## Authors' contributions

WVM is primary responsible for the study as presented in this paper. He made a significant intellectual contribution to the manuscript and has been involved in drafting and revising the manuscript critically.

LE is the executive researcher of the FoodSteps project as presented in this article. He was responsible for preparing and implementing the intervention, collecting and analysing the data and writing of the manuscript.

MVP made substantial contribution to the conception and the design of the study. In addition, MVP has been involved in drafting and revising the manuscript critically.

MCAP made a substantial contribution to the conception and the design of the study, analysing the data, drafting and revising the manuscript critically.

All authors read and approved the final manuscript.

## Pre-publication history

The pre-publication history for this paper can be accessed here:



## Figures and Tables

**Table 1:** Baseline characteristics of the study population

**General characteristics**	**Intervention group *n *= 244**	**Control group *n *= 271**
Gender (% women)	36.9	42.1
Highly educated a (%)	70.8	63.5
Smoking (%)	19.7	15.9
Alcohol consumption/week (median) b	7.0	5.0
Regular visitor to company canteen (%) ^c^	56.1*	36.9
Bringing lunch to work (%) ^d^	43.4	43.2
Diet (%)	4.9	8.9
Mean age (SD)	45.3 (9.6)	45.5 (8.7)
Mean hours per week at the office (SD)	35.3 (5.5)*	36.6 (5.7)
Mean BMI (kg/m^2^) (SD)	26.4 (3.2)	26.5 (2.8)

**Food habits**		

Mean fat intake/day ^e ^(SD)	10.7 (4.1)	10.1 (4.0)
Mean vegetables intake/day (grams) ^f ^(SD)	165.6 (86.3)	149.4 (84.3)
Median fruit (incl. juice) intake/day (pieces)^g^	2.0	2.0

**Psychosocial determinants**		

Mean (SD) attitude (-3, +3)		
Fat	0.6 (1.5)	0.6 (1.5)
Fruit	0.4 (1.9)	0.5 (1.8)
Vegetables	0.7 (1.6)	0.7 (1.6)
Mean (SD) social support (-3, +3)		
Fat	-1.6 (1.4)	-1.5 (1.6)
Fruit	-1.2 (1.5)	-1.2 (1.6)
Vegetables	-1.3 (1.4)	-1.3 (1.5)
Mean (SD) self-efficacy (-3, +3)		
Fat	0.9 (1.6)	1.1 (1.6)
Fruit	1.2 (1.5)	1.4 (1.5)
Vegetables	0.0 (1.7)	0.2 (1.8)
Mean (SD) intention (-3, +3)		
Fat	0.2 (1.8)	0.1 (1.7)
Fruit	-0.1 (1.7)	-0.3 (1.5)
Vegetables	-0.2 (1.5)	-0.2 (1.4)

^a ^University education.

^b ^Number of alcoholic units per week.

^c ^At least once a week purchasing products in company canteen.

^d ^Bringing own lunch to work 5 days of the week.

^e ^fat points per day (all categories, except hot meals).

^f ^grams of vegetables (50 grams = 1 spoon) per day.

^g ^pieces of fruit (1 piece ≅ 125 grams) + glasses of juice a day (1 glass ≅ 150 grams)

* Significant difference (*p *< 0.05) between intervention and control subjects at baseline

**Table 2:** Results of linear regression analyses regarding psychosocial determinants of behavior

**Outcome measure**	**3 months Difference in change (95% CI)**	***p***	**12 months Difference in change (95% CI)**	***p***
**Attitude (-3, +3)**				
Fat	0.21 (-0.05; 0.47)	0.12	-0.31 (-0.05; -0.58)	0.02*
Fruit	0.09 (-0.21; 0.39)	0.55	0.02 (-0.27; 0.30)	0.92
Vegetables	0.23 (-0.04; 0.50)	0.10	0.24 (-0.04; 0.51)	0.09
**Social support (-3, +3)**				
Fat	0.34 (-1.04; -0.60)	0.01*	0.26 (-0.92; -0.46)	0.07
Fruit	-0.11 (-0.28; 0.05)	0.18	-0.12 (-0.28; 0.04)	0.13
Vegetables	0.12 (-0.13; 0.38)	0.32	0.07 (-0.20; 0.34)	0.62
**Self-efficacy (-3, +3)**				
Fat	-0.35 (-0.60; -0.09)	0.01*	-0.44 (-0.70; -0.18)	0.01*
Fruit	-0.12 (-0.37; 0.13)	0.35	-0.16 (-0.42; 0.10)	0.23
Vegetables	-0.10 (-0.38; 0.18)	0.46	0.02 (-0.30; 0.33)	0.89
**Intention (-3, +3)**				
Fat	-0.07 (-0.34; 0.20)	0.61	-0.07 (-0.36; 0.21)	0.60
Fruit	-0.09 (-0.34; 0.17)	0.48	0.05 (-0.22; 0.31)	0.73
Vegetables	0.18 (0.06; 0.43)	0.14	0.01 (-0.25; 0.27)	0.93

^a ^Only crude linear regression model presented: adjusted for baseline value of the outcome measure and group allocation (0 = control, 1 = intervention group). Adjusting for predetermined confounders did not change the results. A positive difference (*diff*.) indicates a change in favor of the intervention group. * *p *< 0.05 level

**Table 3:** Results of linear regression analyses regarding fruit, vegetable and fat intake

**Outcome measure**	**3 months Difference in change^a ^(95% CI)**	***p***	**12 months Difference in change (95% CI)**	***p***
Fruit intake ^b^				
crude	0.96 (0.90; 1.03)	0.23	1.04 (0.97; 1.12)	0.24
adjusted	0.97 (0.91; 1.09)	0.34	1.05 (0.98; 1.12)	0.17
Vegetable intake				
crude	2.8 (-9.0; 14.5)	0.64	1.6 (-9.8; 13.1)	0.78
adjusted	2.5 (-9.4; 14.4)	0.68	1.4 (-10.1; 12.9)	0.24
Fat intake				
crude	0.31 (-0.20; 0.83)	0.23	0.34 (-0.26; 0.93)	0.26
adjusted	0.30 (-0.22; 0.82)	0.25	0.28 (-0.32; 0.88)	0.35
**Subgroup analyses**				
Fat intake & bringing lunch ^c^	-0.25 (-1.02; 0.52)	0.52	-0.08 (-1.00; 0.87)	0.86
Fat intake & not bringing lunch ^d^	0.77 (0.09; 1.45)	0.03*	0.62 (-0.13; 1.37)	0.11

^a ^A positive difference in change indicates a change in favor of the intervention group, except for fat-intake where a negative difference is favorable (= decrease in fat). ^b ^Analyses on fruit intake based on log transformed data. ^c ^Bringing own lunch to work 5 days of the week. ^d ^Bringing lunch to less than 5 days of the week. Crude = linear regression model, adjusted for baseline value of the outcome measure and group allocation (= company). Adjusted = crude regression model, adjusted for gender, BMI, smoking and alcoholic units/wk at baseline. * Significant on *p *= .05 level

## References

[B1] U.S. Department of Health and Human Services (1996). Physical Activity and Health: A Report of the Surgeon General. Atlanta, GA: US Department of Health Human Services, Centers for Disease Control and Prevention, National Center for Chronic Disease Prevention and Health Promotion.

[B2] Blair S, Connelly J (1996). How much physical activity should we do? The case for moderate amount and intensities of physical activity. RQES.

[B3] Pate R, Pratt M, Blair S, Haskell W, Macera C, Bouchard C, Buchner D, Ettinger W, Heath G, King A (1995). Physical activity and public health: A recommendation from the centers for disease control and prevention and the American College of Sports Medicine. JAMA.

[B4] WHO Regional office for Europe (1998). Healthy Nutrition. Preventing nutrition related diseases in Europe. WHO regional publications 24. 1988. Braeckman L, Maes L, Bellemans M, Vanderhaegen M, De Maeyer A, De Bacquer D, and De Backer G, Worker participation in a nutrition education programme. Arch Public Health.

[B5] Hulshof K, Ocke M, van Rossum C, Buurma-Rethans E, Brants H, Drijvers J (2004). Results of the Dutch Food Survey 2003 (article in Dutch) RIVM-rapport nr 350030002.

[B6] Visscher T, Kromhout D, Seidell J (2002). Long-term and recent time trends in the prevalence of obesity among Dutch men and women. Int J Obes Relat Metab Disord.

[B7] Visscher T, Seidell J (2001). The public health impact of obesity. Ann Rev Public Health.

[B8] Seidell J, Visscher T (2003). [Nutrition and health – obesity]. Ned Tijdschr Geneeskd.

[B9] Hu F, Willett W (2003). Optimal diets for prevention of coronary disease. JAMA.

[B10] Dishman R, Oldenburg B, O'Neal H, Shepard R (1998). Worksite Physical Activity Interventions. Am J Prev Med.

[B11] Dunn A, Anderson R, Jakicic J (1998). Lifestyle Physical Activity Interventions. History, Short- and Long-Term Effects and Recommendations. Am J Prev Med.

[B12] Glanz K, Sorenson G, Farmer A (1996). The Health Impact of Worksite Nutrition and Cholesterol Intervention Programs. Am J Health Promot.

[B13] Heaney C, Goetzel R (1997). A review of Health related Outcomes of Multi-component Worksite Health Promotion programs. Am J Health Promot.

[B14] Glanz K, Mullis R (1988). Environmental Intervention to Promote Healthy Eating: A Review of Models, Programs and Evidence. Health Educ Q.

[B15] Wetter A, Goldberg J, King A, Sigman-Grant M, Baer R, Crayton E, Devine C, Drewnowski A, Dunn A, Johnson G, Pronk N, Saelens B, Snyder D, Novelli P, Walsh K, Warland R (2001). How and Why do Individual Make Food and Physical Activity Choices. Nutr Rev.

[B16] Booth SL, Mayer J, Sallis J, Ritenbaugh C, Hill J, Birch L, Frank L, Glanz K, Himmelgreen D, Mudd M, Popkin B, Rickard K, St Jeor S, Hays N (2001). Environmental and Societal Factors Affect Food Choice and Physical Activity: Rationale, Influences, and Leverage Points. Nutr Rev.

[B17] Bracht N (1999). Health Promotion at the community level: new advances.

[B18] Engbers L, Van Poppel M, Chin A Paw M, Mechelen W (2005). Worksite health promotion programs with environmental changes: a systematic review. Am J Prev Med.

[B19] Van Assema P, Brug J, Ronda G, Steenhuis I, Oenema A (2002). A short dutch questionnaire to measure fruit and vegetable intake: relative validity among adults and adolescents. Nutr Health.

[B20] Van Assema P, Brug J, Ronda G, Steenhuis I (2001). The relative validity of a short Dutch questionnaire as a means to categorize adults and adolescents to total and saturated fat intake. J Hum Nutr Diet.

[B21] Vries H, Dijkstra M, Kuhlman P (1988). Self-efficacy: The third factor besides attitude and subjective norm as a predictor of behavioral intentions. Health Education Research.

[B22] Brug J, Lechner L, de Vries H (1995). Psychosocial determinants fruit and vegetable consumption. Appetite.

[B23] Steenhuis I, Van Assema P, Van Breukelen G, Glanz K, Kok G, De Vries H (2004). The impact of educational and environmental interventions in Dutch worksite cafeterias. Health Promot Int.

[B24] Glasgow R, Terborg J, Stryker L, Boles S, Hollis J (1997). Take Heart II, Replication of a Worksite Health Promotion Trial. J Behav Med.

[B25] Sorenson G, Stoddard A, Montange A, Emmons K, Hunt M, Youngstrom R, McLellan D, Christiani D (2002). A comprehensive worksite cancer prevention intervention; behavior change results from a randomised controlled trial (United States). Cancer Causes Control.

[B26] Sorenson G, Stoddard A, Hunt M, Hebert J, Ockene J, Avrunin J, Himmelstein J, Hammond S (1998). The Effect of a health promotion – health protection intervention on behavior change; The Well-Works study. Am J Public Health.

[B27] Sorenson G, Thompson B, Glanz K, Feng Z, Kinne S, DiClemente C, Emmons K, Heimendinger J, Probart C, Lichtenstein E (1996). Work site-based cancer prevention: Primary results from the Working Well Trial. Am J Public Health.

[B28] Emmons K, Linnan J, Shadel W, Marcus B, Abrams D (1999). The Working Healthy Project: A Worksite Health-Promotion Trial Targeting Physical Activity, Diet and Smoking. J Occup Med Environ Health.

[B29] Hebert J, Harris D, Sorenson G, Stoddard A, Hunt M, Hebert J, Harris D, A Stoddard A, Ockene J (1993). A Work-Site Nutrition Intervention: Its effects on the Consumption of Cancer-Related Nutrients. Am J Public Health.

[B30] Jansen J, Schuit A, van der Lucht F (2002). Time for healthy living: health promotion for specific target groups RIVM rapport (in Dutch), ISBN 90-313-38850.

[B31] Larsson I, Lissner L, Wilhelmsen L (1999). The 'Green Keyhole' revisited: Nutritional Knowledge may influence food selection. European Journal of Clinical Nutrition.

